# Supplementary material for: The association between the gut microbiome and 24-h blood pressure measurements in the SCAPIS study
Source: Commun Med (Lond). 2025 Jul 7;5:276. doi: 10.1038/s43856-025-00980-x (PMC12234806; doi:10.1038/s43856-025-00980-x)
Supplement: Supplementary file 2 — Supplementary Information [file 43856_2025_980_MOESM2_ESM.pdf]

Supplementary Information for:

The association between the gut microbiome and 24-hour blood pressure measurements in the SCAPIS study

Yi-Ting Lin, Sergi Sayols-Baixeras, Gabriel Baldanzi, Koen F. Dekkers, Ulf Hammar, Diem Nguyen, Nynne Nielsen, Aron C. Eklund, Georgios Varotsis, Jacob B. Holm, H. Bjørn Nielsen, Lars Lind, Göran Bergström, J. Gustav Smith, Gunnar Engström, Johan Ärnlöv, Johan Sundström, Marju Orho-Melander, Tove Fall

Table of Contents

Supplementary Methods 2

Medication 2

Blood pressure measurements 2

Plasma metabolomics 3

References 4

Supplementary Figures 5

Supplementary Methods

Medication

The participants who had a prescription for antihypertensive medications for the previous 12 months before the visit 1 were obtained from the Swedish Prescribed Medication Register and included the Anatomical Therapeutic Chemical Classification System (ATC) codes C02, C03A, C03EA01, C07, C08C, and C09. Prescriptions for antibiotics for the previous 6 months before the visit 1 included J01CE02, J01CF05, J01EA01, J01FA01, J01FA06, J01FA09, J01FA10, J01FF01, J01XC01, J01XE01, J01AA02, J01AA04, J01AA06, J01AA07, J01CA04, J01CA08, J01CR02, J01DB05, J01DD14, J01EE01, J01MA02, J01MA06, J01MA12, J01MA14, J01XX05, and J01XX08. Participants with prescriptions for narrow-spectrum and/or for broad-spectrum were considered as participants treated with antibiotics.

Blood pressure measurements

Participants wore the ambulatory blood pressure monitoring (ABPM device, Labtech EC-3H/ABP, Labtech Ltd, Debrecen, Hungary) for one day. Systolic blood pressure (SBP) and diastolic blood pressure (DBP) were measured automatically every 30 minutes in the participants from the Malmö center and every 30 minutes during the day and every 90 minutes during the night in participants from the Uppsala center. This nighttime restriction in Uppsala was applied because of a simultaneous sleep registration. Individuals with fewer than 10 readings during daytime or fewer than 5 readings during nighttime were not considered for further analyses. Daytime and nighttime were defined using narrow fixed clock-time periods as 10 AM to 8 PM and 12 AM to 6 AM, respectively, to eliminate the influence of BP variation during the sleep-awake transition.[1] To avoid an overestimation of mean 24-hour BP due to a higher number of readings per hour during daytime, we performed an inverse weighted estimate based on the time interval between measurements.[2,3] Office blood pressure (BP) was measured placing the cuff at the upper arm at the heart level, and the cuff size was adjusted according to individual arm circumference. Measurements were repeated until two subsequent results were within ±10 mmHg with a maximum number of attempts of four. The mean of the two measurements in each arm was used as office BP.

Plasma metabolomics

Metabolomics analysis performed at Metabolon, TX, USA from plasma samples was previously detailed in Dekkers et al.[4] and is summarized here. The processing sequence incorporated randomization of samples alongside various control standards, including blank water samples, extraction reagents, Metabolon's internal human plasma reference material, and a pooled sample from study participants. The sample preparation protocol involved protein extraction through methanol-based precipitation, utilizing a GenoGrinder 2000 system from Glen Mills for aggressive agitation, followed by centrifugal separation. Four distinct analytical approaches were implemented to achieve comprehensive metabolite detection: reversed-phase ultra-performance liquid chromatography coupled with tandem mass spectrometry (RP/UPLC-MS/MS) using negative electrospray ionization, HILIC/UPLC-MS/MS methodology, and two additional RP/UPLC-MS/MS protocols employing positive electrospray ionization. Metabolon's proprietary systems handled peak detection, quantification, and quality assurance procedures. For measurements below detection limits, values were substituted using the lowest detected concentration for each respective metabolite. Compound identification relied on comparisons against Metabolon's extensive reference database containing over 3,300 authenticated standards and unknown substances, utilizing parameters such as retention time indices, mass-to-charge measurements, and chromatographic characteristics. The metabolic classification system assigned two categorical levels to each compound: a broad "metabolite class" designation and a more specific "metabolite subclass" category. The analysis included metabolites with detection frequencies exceeding 100 observations. Metabolites with less than 2% values above the detection threshold were dichotomized. Metabolites classified as drugs by Metabolon were dichotomized, except for 2,6-dihydroxybenzoic acid and De(carboxymethoxy) cetirizine acetic acid which were detected in more than 99% of the individuals.

References

1 Fagard, R., Brguljan, J., Thijs, L. & Staessen, J. Prediction of the actual awake and asleep blood pressures by various methods of 24 h pressure analysis. *J Hypertens* **14**, 557-563 (1996).

2 Mena, L. *et al.* A reliable index for the prognostic significance of blood pressure variability. *J Hypertens* **23**, 505-511 (2005).

3 Octavio, J. A. *et al.* Time-weighted vs. conventional quantification of 24-h average systolic and diastolic ambulatory blood pressures. *J Hypertens* **28**, 459-464 (2010).

4 Dekkers, K. F. *et al.* An online atlas of human plasma metabolite signatures of gut microbiome composition. *Nat Commun* **13**, 5370 (2022).

Supplementary Figures

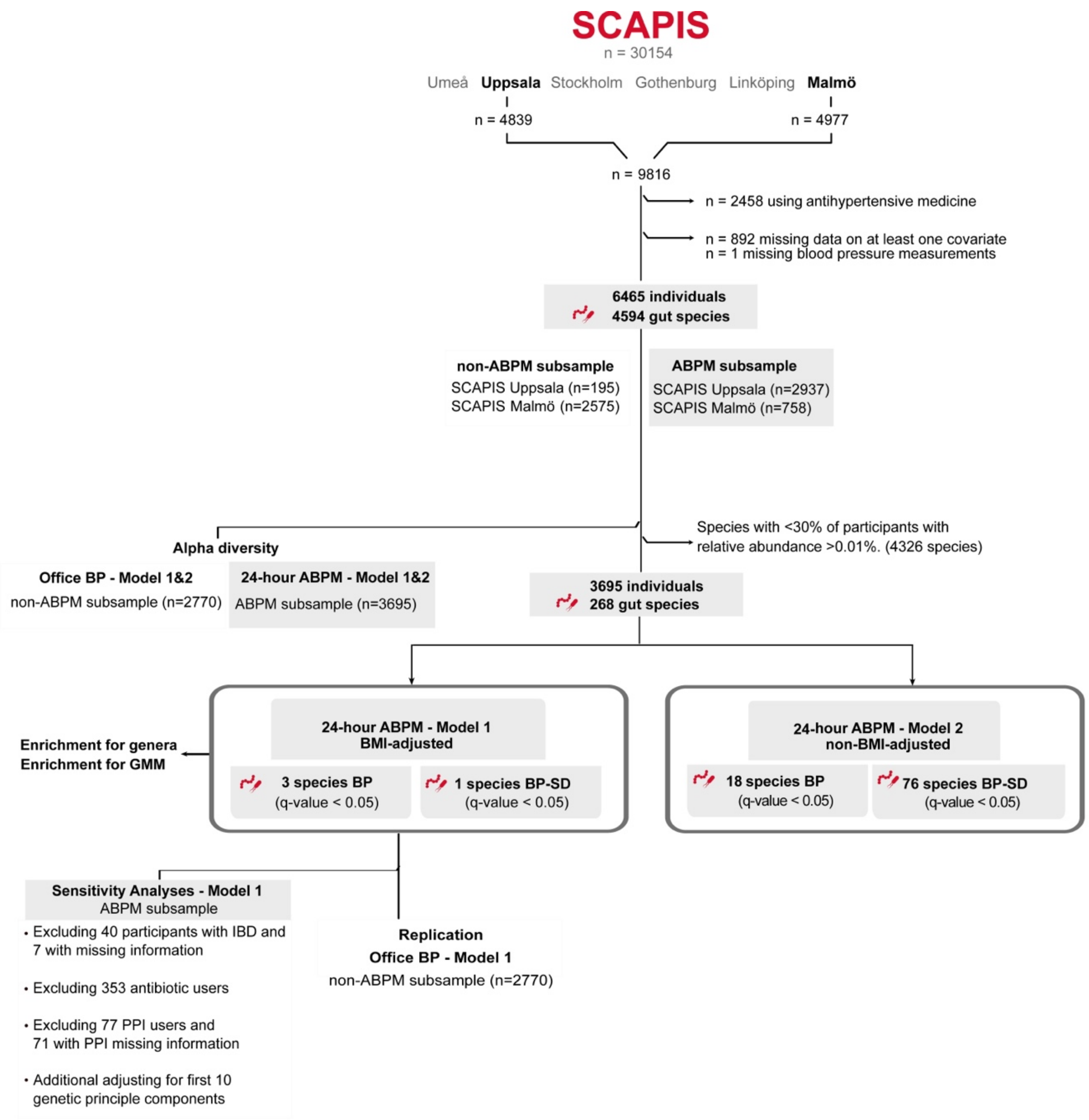

Fig. S1. Flowchart of study design

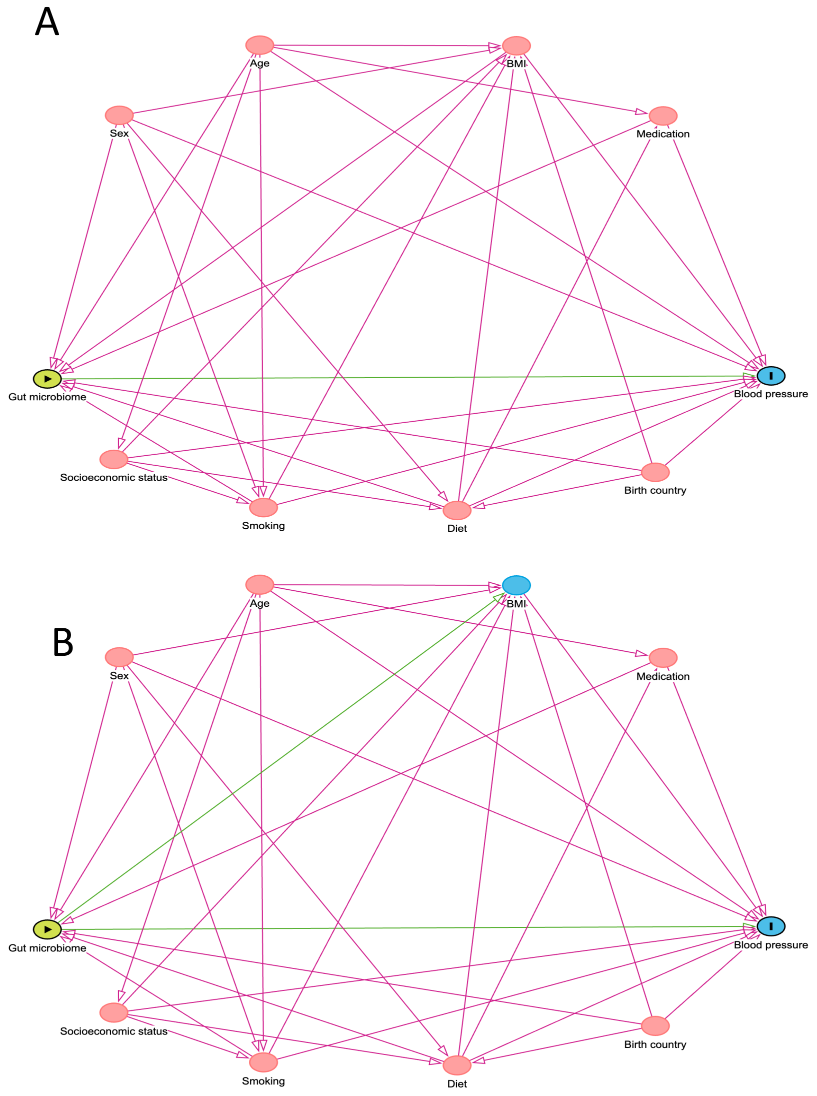

Fig. S2. Directed acyclic graph

Proposed directed acyclic graphs for the association between gut microbiome and blood pressure. (A) Body mass index (BMI) is considered a confounding factor. (B) BMI is considered a mediator. A directed edge (or "arrow") from one node to another represents a direct effect between these two nodes. The exposure (gut microbiome) is denoted by the green oval with the play symbol. The outcome (blood pressure) is denoted by the blue oval with the bar. The causal paths are noted in green lines and the biasing paths are in magenta. Blue oval (empty) represented a mediator. Pink oval represented the potential confounding factors.

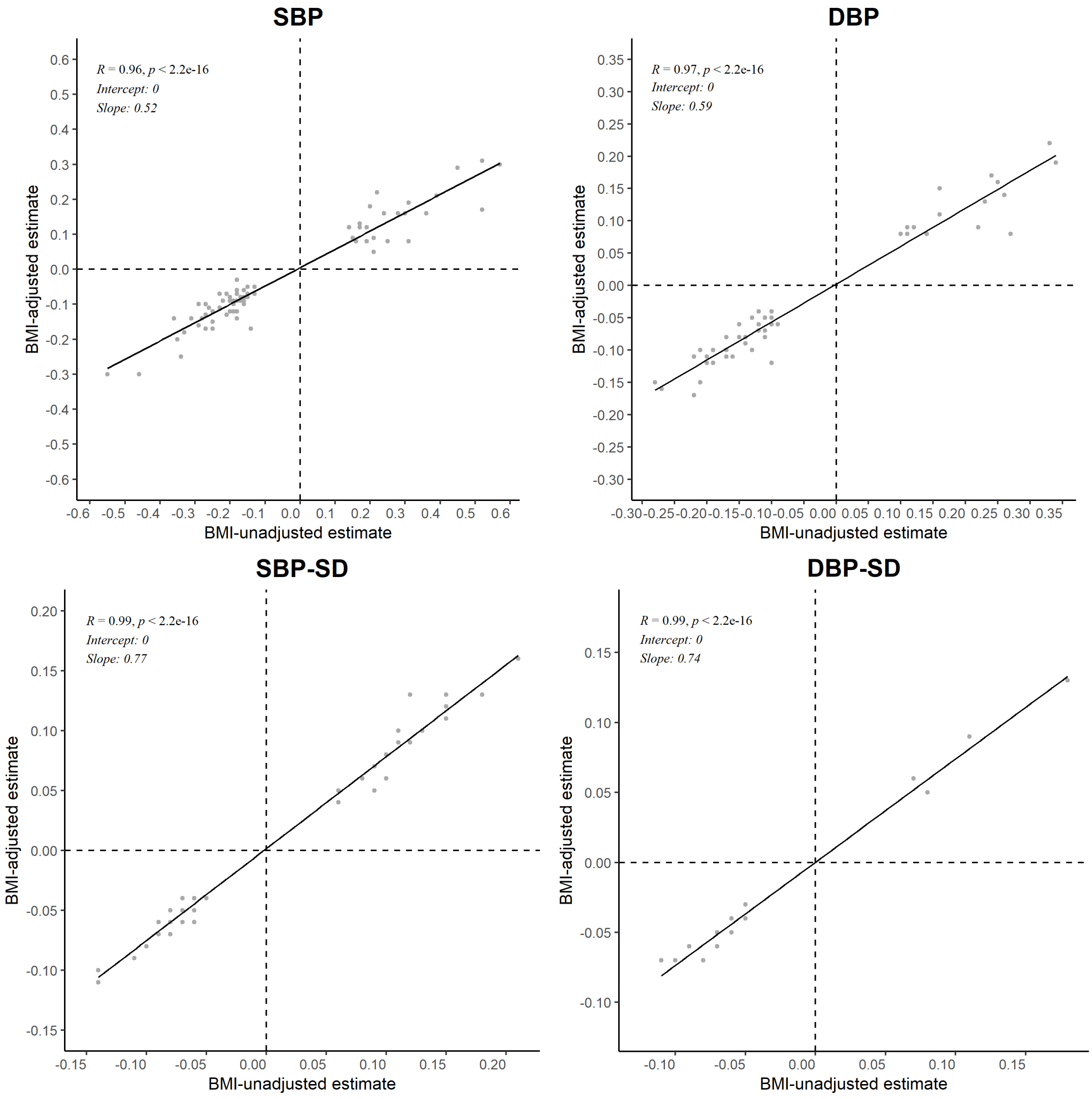

Fig. S3. Scatter plot comparing Model 1 and Model 2

Scatter plots comparing beta estimates from Model 1 (BMI-adjusted) and Model 2 (BMI-unadjusted) for blood pressure-related traits: systolic blood pressure (SBP), diastolic blood pressure (DBP), and variability of systolic blood pressure (SBP-SD) and of diastolic blood pressure (DBP-SD). Each point represents a significant association (q-value <0.05 in Model 2) between a variable and one of the blood pressure traits.

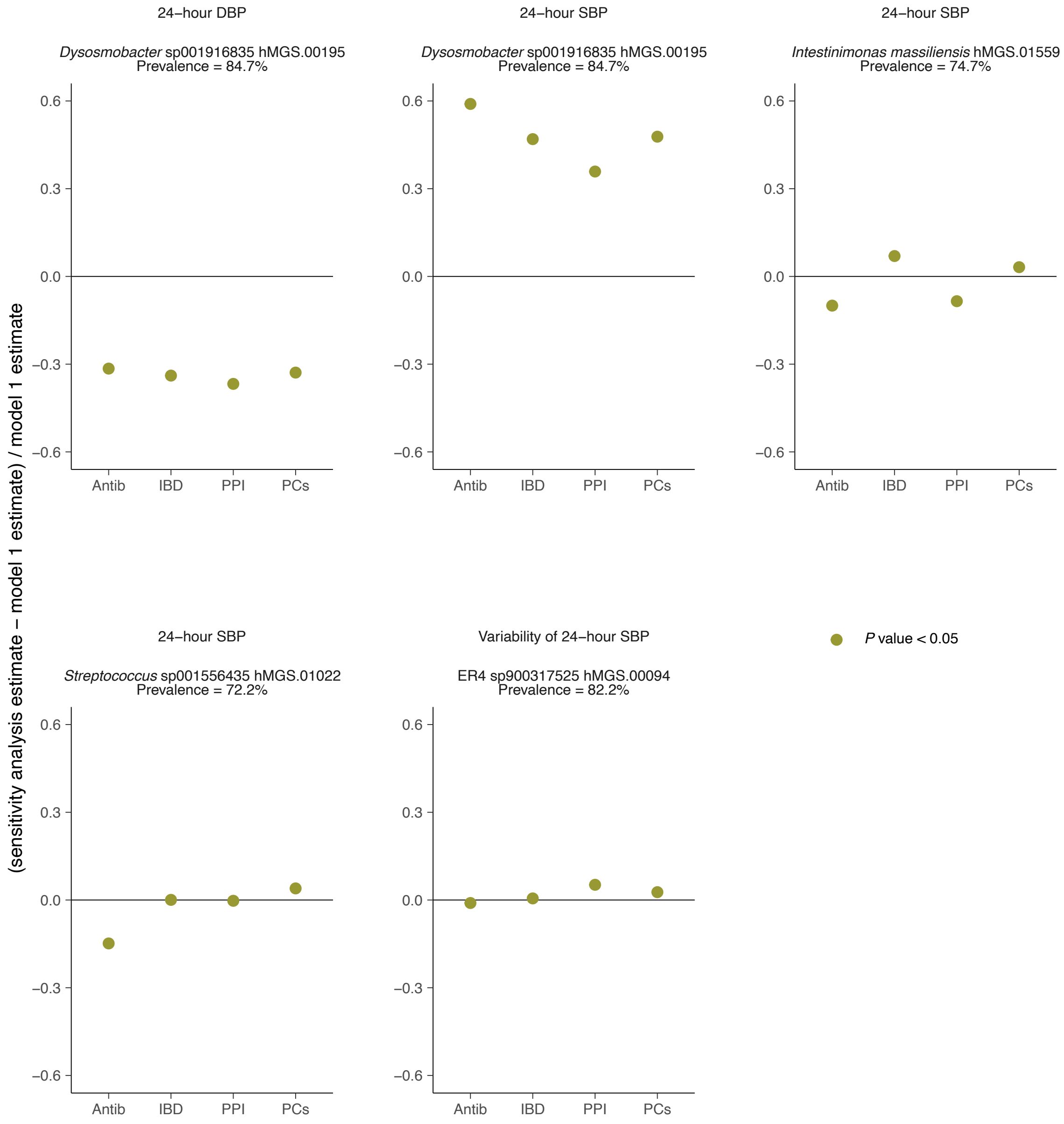

Fig. S4. Sensitivity analyses

Association between results from the Model 1 with 24-hour blood pressure outcomes in which participants who underwent antibiotics treatment (Antib) during the previous 6 months before visit 1, had inflammatory bowel disease (IBD), had proton-pump inhibitors (PPI) measured in plasma, were excluded. Additional adjustments were made for genetic principal components to account for population stratification by adding the first 10 principal components (PCs:1-10).

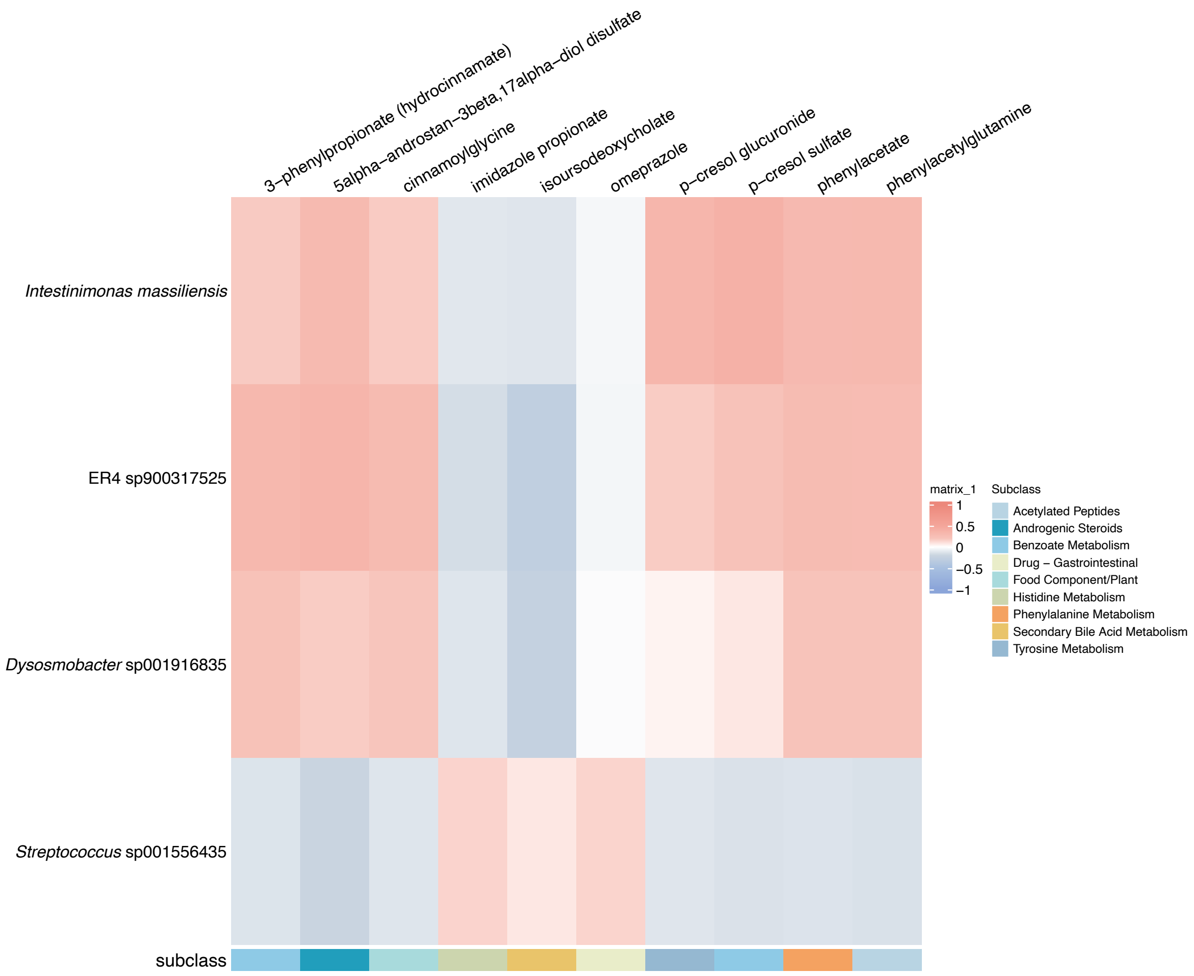

Fig. S5. Heatmap with associations

Heatmap shows associations between 24-hour BP-associated species and the 10 annotated plasma metabolites with lowest *P*-values. The color scale ranges from -1 (dark blue, indicating strong negative correlation) to 1 (dark red, indicating strong positive correlation), with white representing no correlation (0). Metabolites are categorized into distinct subclasses.
