# Supplementary material for: The association between the gut microbiome and 24-h blood pressure measurements in the SCAPIS study
Source: Commun Med (Lond). 2025 Jul 7;5:276. doi: 10.1038/s43856-025-00980-x (PMC12234806; doi:10.1038/s43856-025-00980-x)
Supplement: Supplementary file 3 — Description of Additional Supplementary Files [file 43856_2025_980_MOESM3_ESM.docx]

Description of Additional Supplementary Files

Supplementary Data 1.

Associations between metagenomic species and 24-hour blood pressure outcomes from the 24-hour ABPM subsample in Model 1. Model 1 is adjusted for age, sex, country of birth, smoking, fiber intake, total energy intake, estimated sodium intake, use of antidiabetic medication, use of antihyperlipidemic medication, body mass index, and technical source of variation. Name of species indicates the lowest taxonomic rank available to annotate the species; coef. indicates the regression coefficient of the linear model. All species have been CLR-transformed after adding an offset corresponding to the minimum relative frequency in the dataset.; q-values were generated by applying the Benjamini-Hochberg method to *P*-values for each phenotype. SBP = systolic blood pressure; DBP = diastolic blood pressure; CI = confidence interval. The influential observation analysis excludes the individual with the largest absolute dfbeta value for the exposure.

Supplementary Data 2.

Genera enriched for associations between metagenomic species and 24-hour blood pressure outcomes in the 24-hour ABPM subsample in Model 1. Model 1 is adjusted for age, sex, country of birth, smoking, fiber intake, total energy intake, estimated sodium intake, use of antidiabetic medication, use of antihyperlipidemic medication, body mass index, and technical source of variation. Taxon-set enrichment analysis was applied on the ranked *P*-values from Model 1 for positive and negative regression coefficients separately. Size indicates the number of species belonging to that genus in the analysis, and Coef. indicates the normalized enrichment score; q-values were generated by applying the Benjamini-Hochberg method to the enrichment *P*-values from each phenotype. DBP = diastolic blood pressure; SBP = systolic blood pressure.

Supplementary Data 3.

Gut metabolic modules (GMM) enriched for associations between metagenomic species and 24-hour blood pressure outcomes in the 24-hour ABPM subsample in Model 1. Model 1 is adjusted for age, sex, country of birth, smoking, fiber intake, total energy intake, estimated sodium intake, use of antidiabetic medication, use of antihyperlipidemic medication, body mass index, and technical source of variation. Taxon-set enrichment analysis was applied on the ranked *P*-values from Model 1 for positive and negative regression coefficients separately. GMM level 1 and GMM level 2 are the lowest and the highest hierarchical classification, respectively. Size indicates the number of species belonging to that genus in the analysis, and Coef. indicates the normalized enrichment score; q-values were generated by applying the Benjamini-Hochberg method to the enrichment *P*-values from each phenotype. DBP = diastolic blood pressure; SBP = systolic blood pressure.

Supplementary Data 4.

Associations between the metagenomic species and 24-hour blood pressure outcomes from the 24-hour ABPM subsample in Model 2. Model 2 is adjusted for age, sex, country of birth, smoking, fiber intake, total energy intake, estimated sodium intake, use of antidiabetic medication, use of antihyperlipidemic medication, and technical source of variation. Name of species indicates the lowest taxonomic rank available to annotate the species; coef. indicates the regression coefficient of the linear model. All species have been CLR-transformed after adding an offset corresponding to the minimum relative frequency in the dataset.; q-values were generated by applying the Benjamini-Hochberg method to *P*-values for each phenotype. SBP = systolic blood pressure; DBP = diastolic blood pressure; CI = confidence interval. The influential observation analysis excludes the individual with the largest absolute dfbeta value for the exposure.

Supplementary Data 5.

Sensitivity analysis of the associations between the metagenomic species and 24-hour blood pressure outcomes from the 24-hour ABPM subsample. Participants were excluded if they used antibiotics in the previous 6 months, if they were diagnosed inflammatory bowel diseases, and if they used proton pump inhibitors. Additional adjustments were made for genetic principal components to account for population stratification. The significant results from Model 1 are presented. Model 1 is adjusted for age, sex, country of birth, smoking, fiber intake, total energy intake, estimated sodium intake, use of antidiabetic medication, use of antihyperlipidemic medication, body mass index, and technical source of variation. Name of species indicates the lowest taxonomic rank available to annotate the species; coef. indicates the regression coefficient of the linear model. All species have been CLR-transformed after adding an offset corresponding to the minimum relative frequency in the dataset.; q-values were generated by applying the Benjamini-Hochberg method to *P*-values for each phenotype. SBP = systolic blood pressure; DBP = diastolic blood pressure; CI = confidence interval.

Supplementary Data 6.

Partial Spearman correlations between 4 BP-signature gut microbial species and 1302 plasma metabolites. Correlation coefficients are adjusted for age, sex, country of birth, technical variation, and body mass index.
